# The prevalence of Fabry disease among 1009 unrelated patients with hypertrophic cardiomyopathy: a Russian nationwide screening program using NGS technology

**DOI:** 10.1186/s13023-022-02319-4

**Published:** 2022-05-16

**Authors:** K. Savostyanov, A. Pushkov, I. Zhanin, N. Mazanova, S. Trufanov, A. Pakhomov, A. Alexeeva, D. Sladkov, A. Asanov, A. Fisenko

**Affiliations:** 1 Federal State Autonomous Institution, “National Medical Research Center for Children’s Health” of the Ministry of Health of the Russian Federation, Moscow, Russia; 2 Federal State Autonomous Educational Institution of Higher Education I.M. Sechenov First Moscow State Medical University of the Ministry of Health of the Russian Federation (Sechenov University), Moscow, Russia

**Keywords:** Fabry disease, Selective screening, Hypertrophic cardiomyopathy, NGS, α-gal A, Lyso-Gb3, *GLA*

## Abstract

**Background:**

There is a vast number of screening studies described in the literature from the beginning of the twenty-first century to the present day. Many of these studies are related to the estimation of Fabry disease (FD) morbidity among patients from high-risk groups, including adult patients with hypertrophic cardiomyopathy (HCM) and left ventricular hypertrophy (LVH). These studies show diverse detection frequencies (0–12%) depending on the methodology. Our study is the only example of large-scale selective FD screening based on the implementation of next-generation sequencing technology (NGS) as a first-level test to estimate FD morbidity in the Russian population over 18 years of age burdened with HCM.

**Methods:**

The study included 1009 patients (578 males and 431 females), with a median age of 50 years, who were diagnosed with HCM according to current clinical guidelines. In the first stage of screening, all patients underwent molecular genetic testing (NGS method) of target regions. These regions included the coding sequences of 17 genes and mutations that can lead to the development of HCM. Lysosomal globotriaosylsphingosine (lyso-Gb3) concentrations and α-galactosidase A (α-gal A) enzyme activity were measured in the second stage of screening to reveal pathogenic or likely pathogenic variants in the *GLA* gene.

**Results:**

We revealed 8 (0.8%) patients (3 (37.5%) males and 5 (62.5%) females) with an average age of 59 ± 13.3 years who had pathogenic, likely pathogenic variants and variants of uncertain significance (VUS) in the *GLA* gene (NM_000169.2) as a result of selective screening of 1009 Russian patients with HCM. FD was confirmed via biochemical tests in a male with the pathogenic variant c.902G > A, p.R301Q as well as in two females with likely pathogenic variants c.897C > A, p.D299E and c.1287_1288dup, p.*430Fext*?. These tests showed reduced enzymatic activity and increased substrate concentration. However, a female with the pathogenic variant c.416A > G, p.N139S and with normal enzymatic activity only had increased substrate concentrations. The revealed nucleotide variants and high values of biochemical indicators (lyso-Gb3) in these 4 patients allowed us to estimate the FD diagnosis among 1009 Russian patients with HCM. Mild extracardiac manifestations were observed in these four patients; however, both biochemical values within the reference range in females with the c.971T > G, p.L324W (VUS) variant. α-gal A activity and lyso-Gb3 concentrations were also within the normal range in two males with hemizygous variants, c.546T > C, p.D182D and c.640-794_640-791del (we regarded them as VUS), and in one female with the c.427G > A, p.A143T variant (with conflicting interpretations of pathogenicity).

**Conclusion:**

The prevalence rate of FD among 1,009 adult Russian patients with HCM was 0.4%. We recommend FD screening among adult patients of both sexes with HCM and an undefined genetic cause via NGS method with subsequent analysis of α-gal A activity and lyso-Gb3 concentration in patients with pathogenic, likely pathogenic variants, and VUS. This strategy identifies patients with an atypical form of FD that is characterized by high residual activity of α-gal A, low concentrations of lyso-Gb3, and minor extracardiac manifestations.

## Introduction

Hypertrophic cardiomyopathy (HCM) is a genetic myocardial disease characterized by hypertrophy of the nondilated left ventricle that cannot be explained by secondary causes. It is characterized by significant heterogeneity in morphology, clinical signs, genetic aetiology, and outcomes. In most adult patients, HCM has a relatively benign course; however, in adolescents and young adults, HCM is a frequent cause of sudden cardiac death [43, 53]. Such complications can be prevented via implantable cardioverter defibrillators in high-risk patients. Currently, mutations in more than 30 genes have been identified to lead to HCM. Approximately 50% of familial HCM cases are caused by mutations in the sarcomeric genes *MYH7* and *MYBPC3*, which encode myosin heavy chain and myosin-binding protein C, respectively [1]. Approximately 40% of patients with HCM have no identified mutations [35]. Some scientists associate this with the fact that the causal genes have not yet been identified, while other scientists consider such cases non-Mendelian HCM cases that have a favourable prognosis [9]. Although revealing new genes and mechanisms of the pathogenesis of HCM remains important, the revaluation of Mendelian genetic correlations in HCM indicates that the current clinical genetic testing should be limited to the small number well-established genes with well-understood results [37]. In addition, we should consider that mutations in the genes responsible for several storage diseases can also cause the HCM phenotype, which are referred to as phenocopies.

One such HCM phenocopy is Fabry disease (FD), and one of its manifestations is HCM. FD is an X-linked genetic disease that is caused by pathogenic variants of the *GLA* gene. These variants lead to reduced activity of lysosomal α-galactosidase A (α-gal A) and the intracellular accumulation of neutral glycosphingolipids in various organs and tissues [10].

The classical form of FD is characterized by multisystemic damage and symptoms such as acroparesthesias, proteinuria, keratopathy, angiokeratomas, hypohidrosis, enlarged abdomen, left ventricular hypertrophy (LVH), and hearing loss [28]. FD progression in patients with the classical form may lead to the development of hypertrophic cardiomyopathy, chronic renal failure and stroke [14]. LVH develops in approximately 80% of males and 20–33% of females [26].

In contrast, the atypical form of FD is characterized by damage to one system. Patients with this form of FD have been revealed in screening studies aimed at the diagnosis of patients in high-risk cohorts, such as people with chronic renal failure [38] or cardiomyopathy [45]. In 1990, a number of patients with the atypical form of FD developed isolated LVH, which was similar to sarcomeric HCM [17]. Later, it became known that the development of the FD clinical manifestations is directly associated with residual activity of α-galA [34]. Patients with FD do not always have clinical manifestations that are typical of the classical form due to residual α-galA levels. However, they can manifest later, and the patient could be burdened with HCM or renal failure [7, 58].

The prevalence of FD among patients with LVH [3], patients with HCM [12, 44], and patients with both LVH and HCM [54] has already been estimated by our foreign colleagues. A prevalence ranging from 0 to 12% was reported, depending on the inclusion criteria and diagnostic methodology. Doeney and colleagues reanalysed all screening studies that were published from 1995 to 2017. They summed up all the data and provided more reliable estimations on the prevalence of previously unrecognized FD in cardiac patient cohorts at the 0.9% level in both males and females [15]. Most such screening studies were conducted in adult male patients due to false-negative values of the enzymatic diagnosis of FD in females [23]. However, the biomarker lyso-Gb3 [49] accumulated in the body of FD patients and has been used as an analyte for primary screening in recent years. However, not all patients with elevated levels of this biomarker experienced mutations, despite the proven efficacy of its use in determining the clinical significance of undescribed variants [40]. Moreover, Sanger sequencing of all coding and adjacent intronic regions of the *GLA* gene had been used as the most sensitive method in clinical practice for screening for FD in females [39]. Modern technologies of massive parallel sequencing (NGS) are often used for the simultaneous analysis of the mutations of several genes that can cause diseases with similar phenotypes (for example, diagnosis of Pompe disease and other diseases with primary muscular lesions) [6]. However, currently, there is only one description of screening via NGS for the atypical form of FD in a small cohort of Chinese patients with HCM [57]. In the work of these colleagues, two patients with FD were revealed, which showed a percentage of FD diagnosis among the examined Chinese patients with HCM equal to 0.93%. We selected this technology to conduct selective screening of the atypical form of FD with late onset and other genetic diseases that manifest with HCM. We have used this technology for adults of both sexes, as it is considered the most informative method. The value of such screening programs for patients with HCM is obvious and undeniable because we can significantly relieve their condition via enzyme replacement therapy or chaperone therapy [59].

## Materials and methods

The selective screening of FD was performed on dried blood spots (on filter paper) obtained from 1009 Russian patients of both sexes (males- 578/females- 431) aged 18–79 years (median age of 50 years) with a diagnosis of HCM based on Russian clinical guidelines for HCM [13]. At the first stage of screening, all patients underwent molecular genetic testing of the target regions of the *ACTC1, DES, FLNC, TPM1, TTR, TNNI3, GLA, LAMP2, MYH7, TNNC1, TNNT2, MYBPC3, MYL2, MYL3, PTPN11, PLN, and PRKAG2 genes*. These mutations have been previously described in patients with HCM [48].

Hybridization probes for the NGS panel were designed via the HyperDesign Tool (Roche). They cover all coding and splice regions of the genes listed above and other regions with described pathogenic variants within introns. The size of the panel was ~ 45 kbp.

DNA was extracted from dried blood spots via the phenol–chloroform method. The quality and quantity of DNA was estimated spectrophotometrically via NanoPhotometer N60 (Implen, Germany) and with the Qubit dsDNA HS Assay Kit for the Qubit 3.0 fluorimeter (Invitrogen, USA).

Libraries for NGS were prepared using a KAPA HyperPlus Kit (Roche, USA) according to the manufacturer’s protocol. The DNA fragmentation time was 15 min to achieve an average fragment length of 350 bp. Target enrichment was carried out using KAPA HyperCap hybridization probes (Roche, USA). Massive parallel sequencing was performed on the MiSeq platform (Illumina, USA) with V2 chemistry (500 cycles, paired-end reads). On average, in every run, 31.5 million reads were obtained, of which, 88% had a Phred score higher than Q30.

Bioinformatic analysis was carried out according to the guidelines of GATK Best Practices (https://gatk.broadinstitute.org/). Briefly, raw reads were trimmed using Trimmomatic (version 0.39) [8]. Then, sequence alignment was performed with Burrows–Wheeler Aligner (version 0.7.17) using GRCh37 genome assembly as a reference [30]. Next, duplicate reads were marked using Picard tools and base quality score recalibration (BQSR) was performed. Then, genetic variations (SNPs and indels) were called with HaplotypeCaller using gatk (version 4.1.2). Next, gene annotation was performed with in-house script to annotate variations present in ClinVar, OMIM and HGMD databases. The pathogenicity of variants not previously described was determined using the Alamut program with the built-in software modules SIFT, PolyPhen HDIV, PolyPhen HVAR, Mutation Taster, FATHMM, CADD13, DANN, M-CAP, REVEL, as well as using the ACMG manual. Finally, a filtering process removed variations outside targeted sequences, with a population frequency > 0.5% (gnomAD v2.1.1).

We measured the activity of the α-gal A enzyme and the accumulation of the lyso-Gb3 substrate in the body of patients with FD in the second stage of screening. This screening was performed after revealing pathogenic, likely pathogenic variants, and VUS with global population frequencies not exceeding 0.5% for diseases with recessive inheritance and 0.01% for diseases with dominant inheritance (according to the gnomAD v2.1.1 database).

Measurement of the lyso-Gb3 concentration was performed on a Maxis Impact tandem mass spectrometer (Bruker, Germany) with positive electrospray ionization via HPLC–MS/MS. Chromatographic separation was performed on a 1260 Infinity II LC Systems (Agilent, USA) chromatograph with a Triart C18 50X2.0 (YMC, Japan) column. Mass spectrometric detection was performed in positive ion detection mode using electrospray ionization. The analytical system was calibrated in the range of globotriaosylsphingosine concentrations from 1.0 to 50.0 ng/ml. The analysis time for one sample was 13 min. The resulting data were processed using the built-in Bruker Data Analysis 4.1 software. The cut-off point for the lyso-Gb3 concentration was 1.91 ng/ml.

Measurement of α-galactosidase A activity was performed on a Sciex 3200 Q-TRAP (AB Sciex, USA) tandem quadrupole mass spectrometer via the HPLC–MS/MS method.

The samples were prepared using reagents from the NeoLSD™ MSMS Kit (PerkinElmer, USA). Chromatographic separation was performed on an LC-20ADXR chromatograph (Shimadzu, Japan) with a 059,123 column for reversed-phase HPLC (Thermo Fisher, USA). Ion detection was performed by flow-injection analysis via the MRM monitoring method. The analysis time for one sample was 3 min. The resulting data were processed using the built-in AnalystMD 1.6 software. The cut-off point for the α-galactosidase A activity was 1.89 μmol/l/h.

## Results

Sequence analysis was performed for 1009 Russian patients with HCM. The average read depth of the target regions was 160x. Moreover, 100% of the target was covered with more than 10x. We revealed 332 patients with pathogenic variants, likely pathogenic variants, and VUS after conducting a complete genetic analysis of all target regions of the studied genes. A total of 198 of these patients had pathogenic or likely pathogenic variants in their genome; moreover, 37 patients had minor genetic variants in two or more genes.

Bioinformatics analysis identified 8 (0.8%) patients (3 (37.5%) male and 5 (62.5%) female) with an average age of 59 ± 13.3 years who had genetic variants in the *GLA* gene. This finding corresponds with the mean data (0.9%) of relevant screening programs for FD that were conducted in cardiac patient cohorts for over 20 years [15]. Of the 8 patients in our study, we revealed missense variants in 5 patients. The 6th patient had a deletion in the branch point site, the 7th had a duplication of two nucleotides, and the 8th had a synonymous variant in the canonical splice site. Family segregation analysis was performed for the relatives of three probands,the others were not available for the study. Table 1 describes the *GLA* mutations, enzyme activity and lysoGb3 levels for the eight individuals found by primary screening.

**Table 1:** Russian patients with HCM whose genome contains pathogenic, likely pathogenic variants, and variants with unknown clinical significance in the *GLA* gene

No. of patient	Gender	Age, years	Nucleotide variant, amino-acid variant	Frequency ingnomAD, %	Pathogenicity (ACMG)	Pathogenicity (HGMD)	α-galA, μmol/l/h (Ref ≥ 189 μmol/l/h)	lyso-Gb3, ng/ml (Ref ≤ 191 ng/ml)
71,404	m	51	*c.640-794_640-791del*	n/a	VUS(PM2)	n/d	4.57	1.20
72,609	f	68	c.1287_1288dup,p.*430Fext*?	n/a	pathogenic(PVS1;PS3;PM2)	n/d	**0.88**	**4.60**
74,418	m	45	*c.546T* > *C, p.D182D*	n/a	VUS(PM2;BP7)	n/d	3.86	0.24
74,695	f	69	*c.427G* > *A, p.A143T*	0.051	VUS(PM1;PM2;PP3)	Possibly not pathogenic [CM972773]	5.46	0.47
76,525	m	42	*c.902G* > *A, p.R301Q*	n/a	pathogenic(PS3;PM1;PM2;PP3;PP5)	Fabry disease, atypical variant[CM900111]	**0.22**	**7.40**
78,074	f	68	*c.416A* > *G,p.N139S*	0.019	VUS (PM1;PM2;PP5;BP4;BP6)	Fabrydisease [CM103681]	2.36	**2.15**
78,335	f	69	*c.971T* > *G, p.L324W*	n/a	VUS(PM2; PP3)	n/d	4.25	0.79
80,670	f	49	*c.897C* > *A, p.D299E*	n/a	likely pathogenic(PS3; PM1; PM2; PP3)	n/d	**1.82**	**3.06**

Transcript: NM_000169.2. Abnormal values of biochemical indicators are marked in bold

The pathogenic variant c.416A > G, p.N139S (rs138886989) (previously described in patients with FD [23, 41, 42]) was revealed in a 59-year-old female with asymmetric HCM, heart pain, hypertensive heart disease, normal enzymatic activity and increased substrate concentrations. Her sister had the same pathogenic variant in the heterozygous state, but α-galA activity and the lyso-Gb3 concentration were within normal values.

The likely pathogenic variant c.897C > A, p.D299E (not described) was revealed in a 49-year-old female with concentric HCM, impaired cardiac conduction, reduced α-gal A activity and increased lyso-Gb3 concentrations. However, there is information in the HGMD database that describes the c.897C > G variant, which results in the same amino acid substitution p.D299E in patients with FD [33, 42].

The pathogenic variant c.902G > A, p.R301Q (rs104894828) (previously described in patients with FD, including a typical form [11, 27, 47]) was revealed in a 42-year-old male with concentric HCM, reduced enzyme activity and increased substrate concentrations. This patient had abdominalgia and microalbuminuria in his mild extracardiac manifestations. We managed to perform molecular genetic testing for her ten-year-old daughter, whose genome contained the same pathogenic variant. However, the child did not have any clinical manifestations of FD at the time of the study.

The variant c.971T > G, p.L324W (VUS, not described) was revealed in a 69-year-old female with normal α-gal A activity and a normal lyso-Gb3 concentration. Bioinformatic analysis predicted the pathogenicity of this variant in eight information software modules out of nine. Analysis of variant c.971T > G, p.L324W segregation in the biological relatives of the screened patient along with the estimation of biochemical indicators could help us to clarify its pathogenicity. However, we did not receive any biomaterial from relatives, as the relatives refused to participate in the study.

The variant c.427G > A, p.A143T (rs104894845) was revealed in a 69-year-old female with HCM, and it has been frequently described as a pathogenic variant [22, 52], VUS [16, 19, 34], and likely benign or benign variant [5, 29, 46]. It was also described in the literature as a variant with incomplete penetrance that causes the development of the late form of FD in patients with HCM [56] and as a variant causing isolated HCM [2]. Our study can only confirm the latest description since neither the screened patient nor any of his biological relatives (two daughters of 31 and 35 years of age) with this mutation have any extracardiac manifestations of FD and the measured biochemical indicators were at normal levels.

Duplication c.1287_1288dup, which results in the stop-loss p.*430Fext*? variant, was revealed in a 68-year-old female with HCM, suspected myocardial infarction in anamnesis, reduced enzymatic activity and increased substrate concentration. This mutation was not previously described in the HGMD database. We suggest that this duplication that leads to frameshift may have appeared de novo since neither of the two proband sisters we examined had this nucleotide variant. Moreover, their parents lived to an old age without any clinical signs of FD, including HCM.

We revealed the VUS (ACMG criteria) synonym variant c.546T > C, p.D182D in the canonical splice site at the end of the third exon in a 45-year-old male with HCM without any clinical FD symptoms and normal α-gal A and lyso-Gb3 values. This variant was not previously described in the HGMD and gnomAD (v.2.1) databases. We also revealed the c.640-794_640-791del variant in a 52-year-old male with normal α-gal A enzyme activity and lyso-Gb3 concentration (Table 1). This variant is located deep in the intron close to the pathogenic variant c.640-801G > A, which is described in patients with the late form of FD with HCM [24, 31].

Additionally, we revealed the likely benign variant c.937G > T, p.D313Y (described in HGMD database as a variant with conflicting interpretations of pathogenicity [CM930335]) in 11 patients with HCM. Two males and nine females with this variant had normal values of α-gal A enzyme activity and lyso-Gb3 concentrations.

## Discussion

Starting with the work of Hagège and colleagues [21], biochemical studies of dried blood spots have been successfully used for FD screening programs in patients with HCM for 10 years. It is driven by its transportability and storage capabilities at room temperature for up to 3 weeks without significant loss of enzyme activity. However, some scientists mention that neither enzyme activity analysis nor lyso-Gb3 measurement have adequate specificity to conduct large-scale screening programs on FD [50]. This point of view is supported by facts such as increased substrate concentration in urine in ~ 15% of patients with heart diseases without any mutations in the *GLA* gene. It is also supported by the normal enzyme activity in patients with FD with mild mutations or in females with FD [4] or its false elevation due to any other reasons [20]. This knowledge was the basis for our selective screening of FD in Russian patients with HCM via the NGS method. We included not only the *GLA* gene but also other genes whose pathogenic variants could cause primary genetic HCM [48]. This allowed us not only to identify new cases of FD among patients with HCM but also to detect the cause of the development of sarcomeric and nonsarcomeric HCM in 324 patients that was up to 32.1% of all examined patients. We do not regard the NGS method as excessive due to its cost being comparable to the cost of sequencing of the *GLA* gene via the Sanger method. Apparently, exome sequencing would look redundant in our case, although there are several similar works in the world literature on large patient cohorts. For example, in one of these studies, Pompe's disease was screened under the symptoms of lower limb muscle weakness [25].

**Fig. 1:**
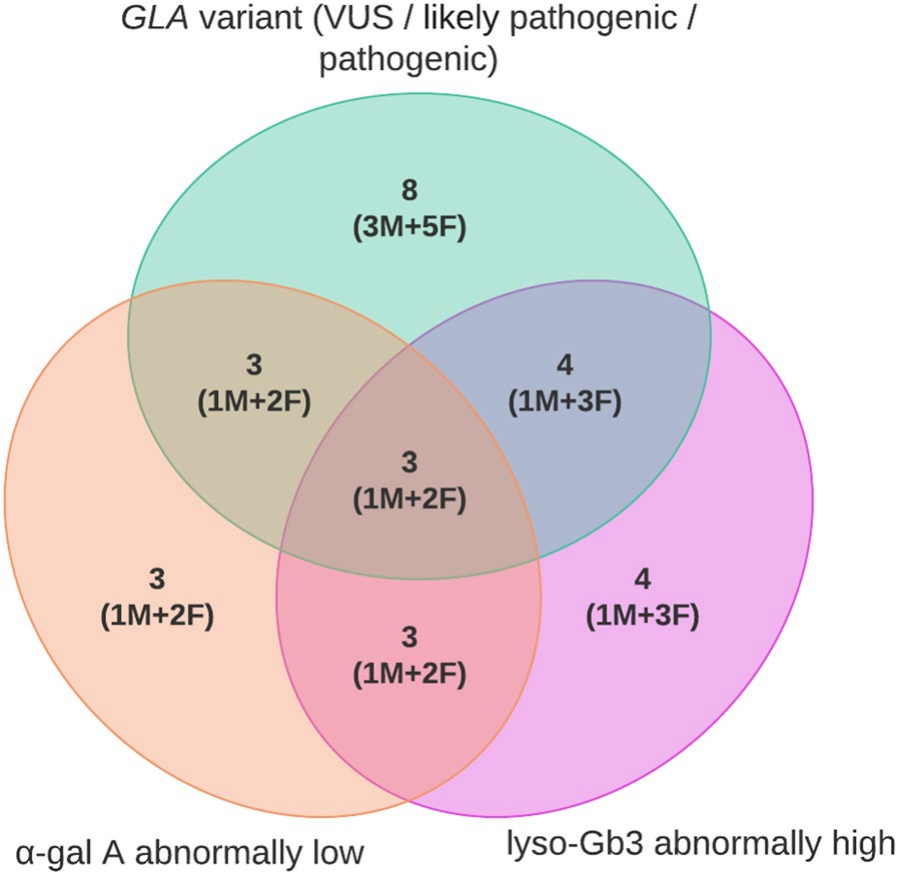
Venn diagram of Russian patients with hypertrophic cardiomyopathy and *GLA* gene variants (pathogenic, likely pathogenic, or VUS), low α-gal A activity and elevated lyso-Gb3 levels in DBS

Screening programs for FD diagnosis in females (including Sanger sequencing) have been successfully used for many years. However, biochemical diagnostics are still very important because they allow us to determine the real number of patients with FD among patients with VUSs [55]. Biochemical analysis was performed in the second stage of our study. It revealed that 4 (0.4%) patients, one male and three females (one of which had normal α-gal A activity), among the 8 (0.8%) patients with pathogenic, likely pathogenic variants, and VUS in *GLA* that were revealed after the first NGS stage had abnormal lyso-Gb3 levels (Fig. 1). There were 2 males and 2 females with rare variants in the *GLA* gene with unknown clinical significance among the 4 remaining patients. The normal levels of lyso-Gb3 and α-galA activity in the males confirm the absence of pathogenicity for the revealed variants and the absence of disease in these patients. However, more studies in females are required to comprehensively interpret the pathogenicity of these variants. There were two females with variants c.427G > A and c.971T > G in the *GLA* gene who had HCM with no extracardial manifestations of FD and normal levels of lyso-Gb3. These two females were not classified as affected females in our study. Thus, they were classified as "carriers of disease". Overlap of the lyso-Gb3 value intervals in affected females with mutations in the *GLA* gene and the control group has been described multiple times in the literature [36]. Moreover, slightly elevated levels of lyso-Gb3 below the cut-off point in females with atypical FD have also been described [51]. According to these data, we can consider that normal lyso-Gb3 levels in females with rare variants in the *GLA* gene cannot exclude FD diagnosis. Furthermore, more evidence is required. For example, family segregation analysis of male relatives is needed to determine the pathogenicity of these variants or measuring the lyso-Gb3 concentration in myocardial biopsy samples is needed in female carriers [24].

Consequently, only additional family segregation analyses can ultimately determine the pathogenicity of c.427G > A and c.971T > G variants and classify these females as patients. The identification of male patients in these two families with the same hemizygous variants and FD diagnosis, confirmed on the biochemical level, would be pertinent.

Moreover, the use of more rigorous inclusion criteria, such as a minimum age of at least 40 years or nondominant inheritance, can lead to an increase in FD definition in cardiac patient cohorts. It can be associated with single cases of myocardial hypertrophy in FD patients before the third decade of life [32], and the dominant inheritance is typical for sarcomeric HCM. Thus, our study supports the idea to identify real FD prevalence among adult Russian patients with diagnosed HCM without any additional inclusion criteria that can exaggerate the results. Therefore, we believe that the FD prevalence in our patient cohort likely reflects the real prevalence of the disease in Russian patients with HCM.

Another argument for the NGS-based approach for the diagnosis of atypical forms of FD is the faster FD diagnosis with further identification of pathogenic variants in burdened families via the Sanger method. It allows the timely prescription of enzyme replacement therapy to prevent cardiovascular complications, such as sudden cardiac death or myocardial infarction, which are common for patients with FD. Overall, our study suggests NGS as a method for FD diagnostics in adult patients with cardiomyopathies and simultaneous diagnosis of the aetiologic causes of genetically determined HCM.

## Conclusion

The FD prevalence among 1009 adult Russian patients of both sexes with HCM was 0.4%. The conducted research has shown the advantages of the NGS method as a first-level test for screening atypical forms of FD in adult patients of both sexes. It also indicates the potential of the targeted genome regions studied within the NGS panel for facilitating the diagnosis of cardiomyopathies. The clinical similarity of the atypical cardiac form of FD and sarcomeric cardiomyopathy suggests that the *GLA* gene (and sarcomeric genes) should be analysed in combined panels used for HCM testing. The existing FD therapy provides pathogenetic care for patients with FD, which in turn can minimize the risk of heart attack and sudden cardiac death and extend life expectancy. However, estimation of α-gal A activity and lyso-Gb3 concentrations are still crucial for the interpretation of the clinical significance of the revealed genetic variants in specific patients.

## Data Availability

Please contact authors for any data requests.
